# Natural history films raise species awareness—A big data approach

**DOI:** 10.1111/conl.12678

**Published:** 2019-09-30

**Authors:** Darío Fernández‐Bellon, Adam Kane

**Affiliations:** ^1^ School of Biological, Earth and Environmental Science (BEES) University College Cork Distillery Fields, North Mall Cork Ireland; ^2^ Environmental Research Institute University College Cork Lee Road Cork Ireland; ^3^ School of Biology and Environmental Science and Earth Institute University College Dublin Dublin Ireland

**Keywords:** big data, conservation message, conservation, David Attenborough, documentary, ecological awareness, information transfer, species awareness, Twitter, Wikipedia

## Abstract

In urbanized societies that are increasingly disconnected from nature, communicating ecological and species awareness is crucial to revert the global environmental crisis. However, our understanding of the effectiveness of this process is limited. We present a framework for describing how such awareness may be transferred and test it on the popular BBC show Planet Earth 2 by analyzing Twitter and Wikipedia big data activity. Despite lacking explicit conservation themes, this show generated species awareness, stimulating audience engagement for information at magnitudes comparable to those achieved by other conservation‐focused campaigns. Results suggest that natural history films can provide vicarious connections to nature and can generate durable shifts in audience awareness beyond the broadcast of the show—key factors for changing environmental attitudes. More broadly, this study underscores how open‐source big data analysis can inform effective dissemination of ecological awareness and provides a framework for future research for investigating behavioral change.

## 1 INTRODUCTION

We live in the Anthropocene, a period when human actions are having a profound effect on our planet (Lewis & Maslin, 2015). Mounting evidence points to the need for immediate action to curb species extinctions, warming temperatures, diminishing natural resources, etc. (Ripple et al., 2017). The urgency of this global crisis contrasts with the slow pace of change in societal awareness and attitudes, and the difficulty in reaching international environmental agreements (Scheffer, Westley, & Brock, 2003; Schleussner et al., 2016). There have, however, been some success stories, such as reductions in CFC emissions and international whale hunting bans (Cunningham, Huijbens, & Wearing, 2012; Parson, 2003) that suggest changes in societal attitudes are not inherently slow. The current environmental crisis results from maladaptive human behaviors (Maloney & Ward, 1973) and requires widespread behavioral changes (IPBES, 2019; Mascia et al., 2003). However, the role of conservation messaging and information transfer in eliciting behavioral changes is still poorly understood (de Lange, Milner‐Gulland, & Keane, 2019; Kidd et al., 2019).

One explanation for the public's apparent disinterest in urgent environmental issues is the “extinction of experience” (Pyle, 1993). Dwindling connections to nature in increasingly urbanized populations lead to a disaffection toward the natural world, a loss of environmental awareness, and a reduction of changes in attitude and behavior (Soga & Gaston, 2016, 2018; but see Hopper, Gosler, Sadler, & Reynolds, 2019; Kudryavtsev, Krasny, & Stedman, 2012 for examples of education‐ and culture‐induced behavioral changes in urban settings). Environmental campaigns have recognized this, and increasingly focus on emotional values to reengage the public (Wu & Lee, 2015). In this context, natural history films have emerged as a platform for reconnecting people to nature, captivating large audiences with entertaining and emotionally charged storylines (Fortner & Lyon, 1985; Sullivan, 2016), but the value of such vicarious connections with nature has been questioned (Ballouard, Brischoux, & Bonnet, 2011; but see Soga, Gaston, Yamaura, Kurisu, & Hanaki, 2016). Moreover, natural history films are often criticized for portraying unrealistic, pristine views of nature (Beck, 2010), despite often featuring endangered species whose survival is time‐sensitive (see for example the collapse of Saiga antelope *Saiga tatarica* populations, witnessed—but not portrayed—by film crews; Kock et al., 2018; Milner‐Gulland, Morgan, & Kock, 2016; Nicholls, 2015). In response to criticism for side‐stepping conservation issues (Monbiot, 2018; Mortimer, 2017), natural history film producers, like David Attenborough, argue that explicit conservation messages are a turn‐off for viewers (Watts, 2018).

**Figure 1:**
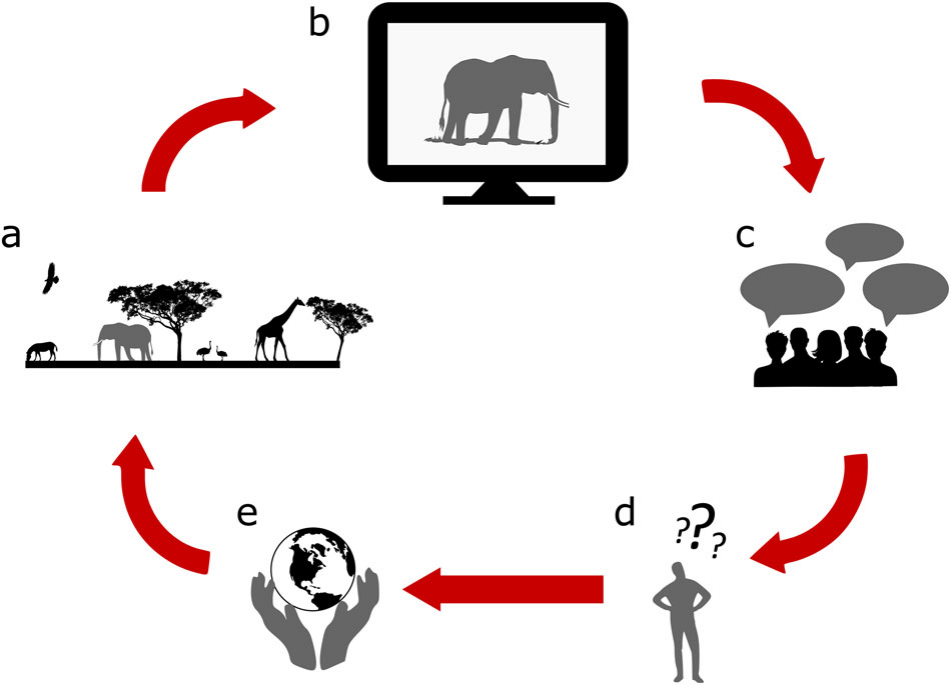
Proposed framework for the dissemination and transfer of ecological awareness. (a) Current knowledge of the natural world and conservation issues affecting it (i.e., ecological and species awareness, conservation status and threats); (b) filtering or selection of this knowledge for dissemination; (c) public reaction; (d) public engagement for information; (e) changes in societal awareness and proactive actions that can feed back into the natural world. In our case study these steps are: diversity and conservation status of wild populations; natural history films; Twitter activity; Wikipedia searches; longer‐term changes in Wikipedia searches and donations to charities

Despite the importance of this debate, only a few studies (e.g., Silk, Crowley, Woodhead, & Nuno, 2018) have quantified audience responses to movie portrayals and documentary representations of the natural world and these have often relied on experimentally controlled approaches (Jacobson et al., 2018; Jain et al., 2019). Here we develop a conceptual framework of how such representations can promote ecological and species awareness and support for the natural world, using natural history films as a case study (Figure 1). We tested our framework against the natural history film series Planet Earth 2 using a novel big data approach. The show was broadcast by the BBC in 2016 and achieved record viewership figures (c. 12 million viewers per episode; Furness, 2016), but was criticized for its unrealistic portrayal of the natural world (Hughes‐Games, 2017; Mortimer, 2017).

We performed qualitative and quantitative analysis of the show's content and analyzed Twitter and Wikipedia big data activity to evaluate audience reactions to the broadcast and subsequent engagement for information (Okoli, Mehdi, Mesgari, Nielsen, & Lanamäki, 2014; Toivonen et al., 2019). As messages delivered through television and other media are considered transient (de Lange et al., 2019), we then investigated whether the show instigated longer‐term changes in public awareness (i.e., beyond the broadcast of the show) and proactive behaviors (Nilsson, Fielding, & Dean, 2019). Finally, we compared the magnitude of the effects of Planet Earth 2 on audiences to that achieved by wildlife awareness campaigns.

## 2 METHODS

### 2.1 Portrayal of the natural world: Planet Earth 2

Six episodes of Planet Earth 2 were broadcast in the United Kingdom from November 6 to December 11, 2016. We performed a qualitative analysis of the script for each episode to identify conservation themes. Both authors independently reviewed the script and highlighted sections concerning environmental and conservation issues. We identified all animal species mentioned in the script (n = 113) and classified them as mammal, bird, reptile, amphibian, fish, or invertebrate. For each species we recorded the total time on screen per episode. As the portrayal of a species might affect public reaction and engagement, we noted whether each species was depicted as part of a predator–prey interaction or in another ecological role. We also collated data on IUCN conservation status (critically endangered, endangered, vulnerable, near threatened, least concern or data deficient) for each species (IUCN, 2017). To understand how the show portrayed the natural world, we collated information on the diversity in the wild (number of species) of each taxonomic group and the proportion of these ascribed to each IUCN conservation status (Chapman, 2009; IUCN, 2017).

### 2.2 Audience reaction: Twitter activity

We used Twitter to measure audience reactions to the content shown in Planet Earth 2 (Toivonen et al., 2019). For each episode we collated tweets using the hashtag #PlanetEarth2 for 2 hr beginning at the start of the episode. For each episode we sampled the first 5,000 original tweets from UK users (excluding retweets) using the online software TweetDeck. To evaluate audience reaction to conservation and environmental topics, we counted the number of tweets mentioning the different conservation themes identified in our qualitative analysis of the script (out of a total of n = 30,000 tweets). To evaluate audience reaction to the species, we counted the number of species mentions in the tweets from the episode each species was featured in (n = 5,000).

We used a negative binomial GLM from the *MASS* library in R to test if time on screen (in seconds) was a significant predictor of number of Twitter mentions for each species. Although we hypothesized that species’ time on screen would have the strongest effect on audience response, we also considered the potential of the following variables to affect Twitter mentions: taxonomic group, IUCN status, species interaction (predator–prey or other) and occurrence in diaries. We assessed model fit by examining diagnostic plots. The multiple regression model with all variables was compared to the simple negative binomial GLM of number of tweets ∼ time on screen using AIC.

### 2.3 Audience engagement: Wikipedia page visits

Beyond initial reactions to the show, we considered audience interest and searches for further information as a form of engagement. Wikipedia is the most popular online encyclopedia and an indicator of public interest in a range of topics (Okoli et al., 2014). We used the R package *pageviews* to find the daily number of hits the Wikipedia page for each species featured on Planet Earth 2 received during 2016.

To measure the impact of Planet Earth 2 on audience engagement we evaluated whether visits to Wikipedia pages of each species increased after the broadcast of the show. We used the R package *AnomalyDetection* to pick out anomalies in the yearly time series data for 2016. We set sensitivity at 1%, meaning there could be a maximum of three anomalies for a page/species over the 365‐day series (although there could be fewer or no anomalies). We counted how many anomalies coincided with the day of the broadcast or the day after broadcast of the episode that featured the corresponding species. To evaluate whether anomaly rates could be expected randomly or were indeed a result of the broadcast of Planet Earth 2, we compared them to 2016 anomaly rates for “control species” featured in Planet Earth 1 (broadcast in 2001).

To determine what factors influenced how many visits a species’ Wikipedia page received, we again used a negative binomial GLM. As a response variable we used the number of page visits that we attributed to Planet Earth 2. For this we first calculated the maximum number of daily visits to each page in the two days following broadcast. Then we calculated the species’ baseline popularity as the median number of Wikipedia page visits received from July 1, 2015 to June 30, 2016 for each page, which we considered to be sufficiently before the influence of Planet Earth 2. The response variable for the GLM was the difference between the maximum number of page visits on broadcast days and the species’ baseline popularity. Similar to our audience reaction analysis (Twitter), we hypothesized that time on screen per episode would have the most significant effect on Wikipedia page visits. We also built a multiple regression with time on screen, taxonomic group, IUCN status, presence in diaries, and species interaction (predator–prey or other). We again assessed model fit by examining diagnostic plots and compared the simple model (negative binomial GLM of the number of Wikipedia article hits corrected for baseline popularity ∼ time on screen) to the multiple regression model using AIC.

### 2.4 Changes in awareness: Long‐term trends in Wikipedia page visits

For those species that registered an anomaly in Wikipedia page visits during the broadcast, we evaluated whether there were longer‐term changes in awareness (this analysis was performed for 43 out of the 46 species that registered anomalies, as there were gaps in Wikipedia page visit time series for 3 species). We built Bayesian structural time‐series models using the R package *CausalImpact* on time series of Wikipedia page visits (Brodersen, Gallusser, Koehler, Remy, & Scott, 2015). The model takes a target time series and compares it to multiple related controls in a period before any intervention—here before the broadcast of Planet Earth 2. It uses this comparison to predict a counterfactual scenario, that is, how the target time series would have behaved in the absence of Planet Earth 2. The difference between the predicted counterfactual time series and the data is a measure of the impact of Planet Earth 2.

### 2.5 Proactive engagement: Conservation charities

We collected daily mean‐standardized donation data for two nature charities, Arkive and the Born Free Foundation for 2016 to test whether the broadcast of Planet Earth 2 resulted in proactive audience engagement with conservation issues. Arkive is an online repository of media of the natural world, with a particular focus on photographs and videos of endangered species, of which David Attenborough is a patron and active promoter, while the Born Free Foundation is one of the leading wildlife charities in the United Kingdom. We focused on these charities as their relevance in the context of Planet Earth 2 (connection with David Attenborough and UK audiences) increased our chances of detecting any impacts from the broadcast of the show. We ran an anomaly detection analysis similar to that used for our Wikipedia analysis with a 1% sensitivity on donation time series for both charities, to evaluate whether any anomalies overlapped with the broadcast of Planet Earth 2 episodes.

### 2.6 Signal strength

To evaluate the magnitude of the effect of Planet Earth 2, we compared audience engagement for information stemming from Planet Earth 2 to that generated by world species awareness days. World species days provide an ideal comparison, as they are conservation‐focused campaigns of a temporal nature similar to that of a natural history film broadcast. For 16 world species days during 2016, we calculated the number of Wikipedia page visits on the campaign date controlling for baseline popularity (i.e., number of page visits on campaign date – median number of page visits).

Further details on methods and code for analysis are available in the supporting information.

## 3 RESULTS

### 3.1 Portrayal of the natural world: Planet Earth 2

**Figure 2:**
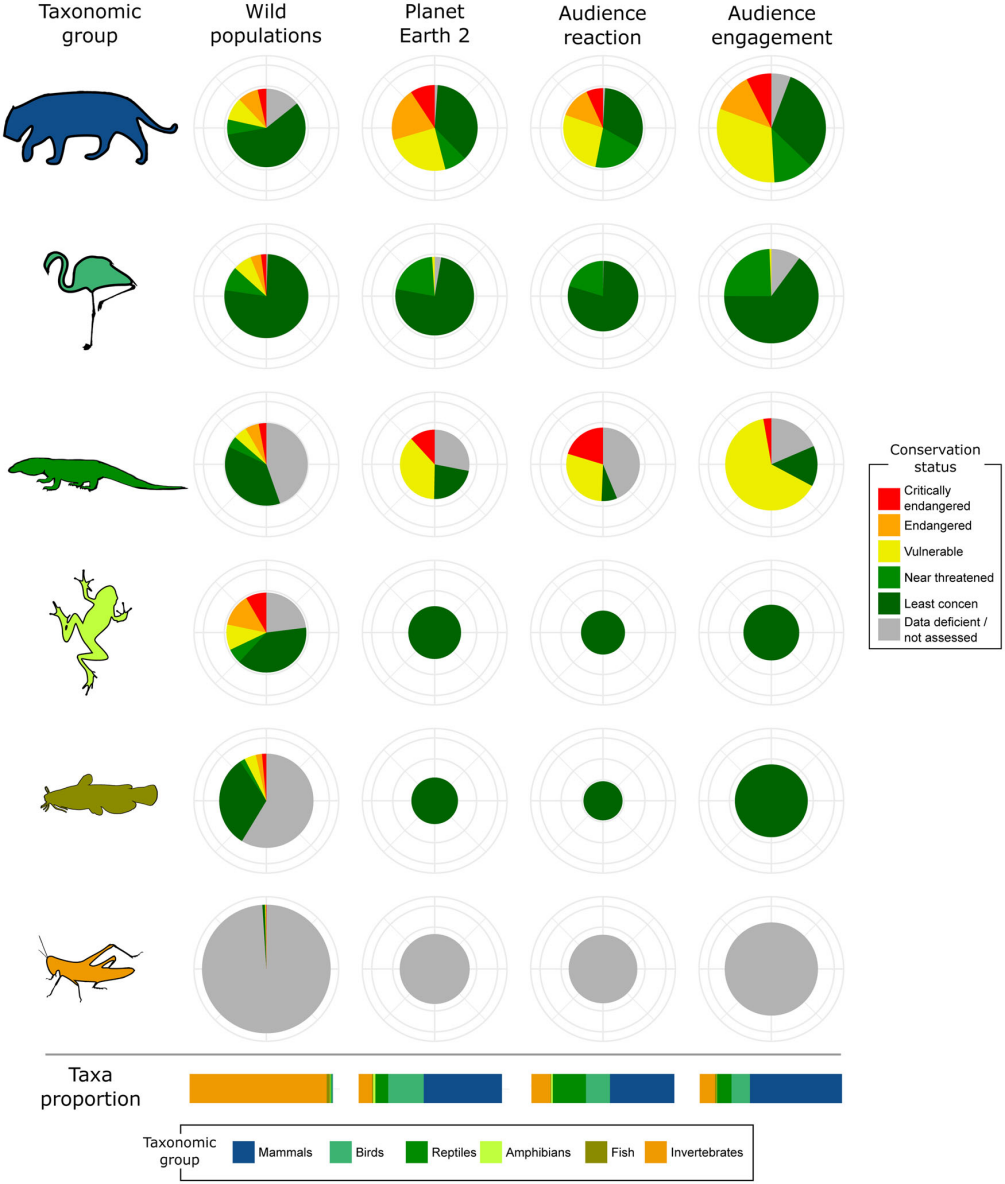
Proportion of taxonomic groups and conservation status across the different steps of our framework. Relative proportion of each taxonomic group (number of species) is represented by circle size (logarithmic scale), and by bars at the bottom of the figure (untransformed scale). Circle colors represent the number of species of different IUCN conservation categories within each group and bar colors represent taxonomic groups. Changes in circle size along columns show relative taxa proportions within each step of the framework (e.g., more invertebrate than mammal species in the wild) and changes in circle size along rows indicates how these proportions change across the framework (e.g., invertebrates underrepresented in Planet Earth 2)

In line with the criticism of Planet Earth 2 (Hughes‐Games, 2017; Mortimer, 2017), we found that only 6% of the script was dedicated to conservation themes and that these had a negligible effect on audience reaction (less than 1% of all tweets mentioned conservation themes, see Tables S1 and S2). As an explicit conservation message was absent from the show, we focused on the animal species portrayed in each episode. We found that mammals were overrepresented in the show, while all other groups were underrepresented (Figure 2, changes in circle sizes), and that time allocated to species of different IUCN categories did not reflect conservation priorities (Figure 2, changes in color proportions).

### 3.2 Audience reaction: Twitter activity

**Figure 3:**
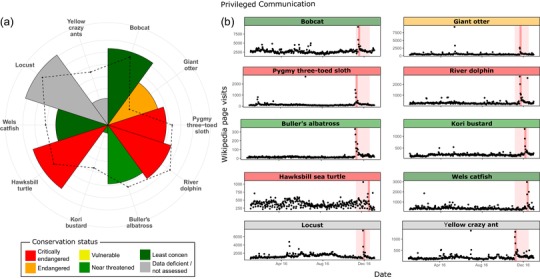
Audience reaction and engagement for ten of the species featured in Planet Earth 2, highlighting how reaction and engagement were driven by time on screen but were independent of taxa or conservation status. (a) Audience reaction was measured as number tweets with the hashtag #PlanetEarth2 mentioning each species; dotted line represents time on screen for each species. Number of tweet mentions and time on screen are log‐transformed (untransformed value ranges: 2–1,250 tweets, 9–779 seconds). (b) Audience engagement was measured as visits to the Wikipedia species pages; red shading indicates the 6 weeks of Planet Earth 2 broadcast and darker red band highlights the broadcast of the corresponding episode

Our models of audience reaction to the show using tweets containing the hashtag #PlanetEarth2 (n = 5,000 tweets per episode) indicated a significant positive effect of species screen time per episode on audience reaction (Figures 3a and S1, Table S3).

### 3.3 Audience engagement: Wikipedia page visits

Our anomaly analysis on Wikipedia time series indicated that 41% of the species featured in the show registered yearly peaks in page visit numbers on broadcast days, significantly more than for “control species” not featured in the show (Figures 3b and S2 and Table S4). The regression models evaluating factors influencing Wikipedia visits showed that species’ time on screen per episode had a significant positive effect on engagement for information (Table S5).

### 3.4 Changes in awareness: Long‐term trends in Wikipedia page visits

Our investigation of long‐term changes in audience awareness showed that 44% of Planet Earth 2 species that had registered anomalies during the broadcast had significantly higher rates of Wikipedia visits 6 months after the show than expected by random patterns (i.e., compared to “control species,” Figure S3 and Table S6).

### 3.5 Proactive engagement: Conservation charities

Anomaly analysis of donations to the two selected charities, BornFree and Arkive indicated no clear relationship between the show and donations during the broadcast dates (Figure S4).

### 3.6 Signal strength

We found that engagement for information (i.e., Wikipedia page visits) following Planet Earth 2 was comparable in magnitude to that generated by “world species days” (Figure S5 and Table S7).

## 4 DISCUSSION

**Figure 4:**
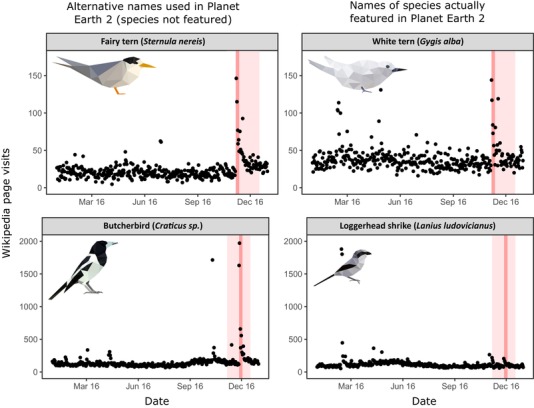
Effects of naming accuracy on audience engagement for information. Two species featured in Planet Earth 2 (*Gygis alba*, commonly known as white tern, and *Lanius ludovicianus*, commonly known as loggerhead shrike (Clements et al., 2018; IUCN, 2017; Wikipedia, 2019) were referred to as “fairy tern” and “butcherbird.” Searches for information of these alternative names used in the show led audiences to other species. In the case of the tern, taxonomic proximity allowed audiences to redirect to the correct Wikipedia page, and both species registered similar peaks. In the second case, however, the page for a different group of birds known as butcherbirds registered a substantial peak, while the species actually featured in the show (loggerhead shrike) did not. This exemplifies how apparently minor details at the dissemination stage can impact species awareness

Our findings suggest that natural history films focused on entertainment can generate vicarious connections with the natural world and increase public interest in nature at both immediate and longer‐time scales. Such vicarious interactions can positively affect a person's interest and attitudes toward environmental issues (Soga et al., 2016). Although focused on entertainment, these films attract wider audiences than shows with an overt conservation agenda whose audiences are likely already environmentally aware (Gross, 2018). In the case of Planet Earth 2, conservation themes were largely absent and generated negligible impact on audiences. Audience reaction (Twitter) and engagement (Wikipedia) to the species featured in the show were driven by time on screen, were independent of taxa and not limited to charismatic megafauna (e.g., locust, railroad worm and crazy ant all recorded peak visits to their Wikipedia pages during the broadcast), and reached magnitudes comparable to those generated by dedicated conservation campaigns (although effects can be hampered by information accuracy, see Figure 4). This result shows how filmmakers or conservation agencies can highlight a particular species or conservation issue by dedicating more airtime to the topic.

The lack of proactive actions stemming from Planet Earth 2 through charitable donations was not completely unexpected, as awareness is just one of many factors influencing these behaviors (Lundberg et al., 2019). Proactive actions in response to conservation messages are the result of complex social processes (de Lange et al., 2019) and may happen at a considerable lag, making it difficult to establish a cause‐and‐effect relationship. Furthermore, there is arguably not a single, dominant nature charity where we would expect a clear signal of public donations (compared to the well‐established proxies we used for audience reaction and engagement).

Although television and other mass media are generally considered to provide transient messages (de Lange et al., 2019), we found that effects on audience awareness of species persisted beyond the broadcast of Planet Earth 2. For shows with a clearer conservation agenda, such longer‐term effects may indeed be linked to public behavioral changes and proactive actions. Furthermore, natural history films coupled with opinion leaders (e.g., David Attenborough), using broader reaching channels (e.g., online streaming platforms), or that engage with the public (e.g., social media campaigns) have strong potential to promote pro‐conservation behaviors (de Lange et al., 2019). Our framework provides an approach that producers and conservationists can use to monitor and inform the transfer of ecological awareness, not only through natural history films, but also through other platforms such as world species days, social media conservation campaigns, online outreach videos, podcasts, etc.

Understanding how to modify societal attitudes is essential if we are to revert the current global environmental crisis. Recent technological developments place us in a unique situation, whereby it is now possible to explore public opinions, interests and attitudes of vast sample sizes by collating different types of online big data. While our approach is not without its limitations (e.g., skewed representation of society, language and cultures, superficiality of information), by incorporating big data it contributes to current understanding stemming from experimental and survey‐based studies. Many tools that could further develop our framework (e.g., big data analysis, text mining, causal impact) are already used in other fields such as market analysis and econometrics (Brodersen et al., 2015), but they remain largely unexplored in the context of conservation.

## DATA ACCESSIBILITY

All data and code are available in the main text, supporting information and online at https://github.com/kanead/documentary-paper.

## Supporting information

FIGURE S1 Audience reaction to the species featured in Planet Earth 2
FIGURE S2 Audience engagement to species featured in Planet Earth 2
FIGURE S3 Causal impact of Planet Earth 2 on long‐term audience awareness
FIGURE S4 Anomaly analysis of charity donation time series
FIGURE S5 Audience engagement for information in response to Planet Earth 2 and to world species days
TABLE S1 Mentions of conservation and environmental themes in Planet Earth 2
TABLE S2 Audience reaction to conservation and environmental themes covered in Planet Earth 2
TABLE S3 Output of negative binomial GLM models explaining audience reaction to Planet Earth 2
TABLE S4 Planet Earth 2 species Wikipedia page visit anomalies
TABLE S5 Output of negative binomial GLM models explaining audience engagement following Planet Earth 2
TABLE S6 Summary statistics of causal impact of Planet Earth 2 on long‐term audience awareness
TABLE S7 Comparison of audience engagement for information in response to Planet Earth 2 and to world species days

Supporting Information
