# Supplementary material for: Natural history films raise species awareness—A big data approach
Source: Conserv Lett. 2019 Sep 30;13(1):e12678. doi: 10.1111/conl.12678 (PMC7074017; doi:10.1111/conl.12678)
Supplement: Supplementary file 1 — FIGURE S1 Audience reaction to the species featured in Planet Earth 2 FIGURE S2 Audience engagement to species featured in Planet Earth 2 FIGURE S3 Causal impact of Planet Earth 2 on long‐term audience awareness FIGURE S4 Anomaly analysis of charity donation time series FIGURE S5 Audience engagement for information in response to Planet Earth 2 and to world species days TABLE S1 Mentions of conservation and environmental themes in Planet Earth 2 TABLE S2 Audience reaction to conservation and environmental themes covered in Planet Earth 2 TABLE S3 Output of negative binomial GLM models explaining audience reaction to Planet Earth 2 TABLE S4 Planet Earth 2 species Wikipedia page visit anomalies TABLE S5 Output of negative binomial GLM models explaining audience engagement following Planet Earth 2 TABLE S6 Summary statistics of causal impact of Planet Earth 2 on long‐term audience awareness TABLE S7 Comparison of audience engagement for information in response to Planet Earth 2 and to world species days [file CONL-13-e12678-s001.docx]

Supporting Information for

Natural history films raise species awareness - a big data approach

Darío Fernandez Bellon*, Adam Kane

*Corresponding author. Email: d.fernandezbellon@umail.ucc.ie

**Supporting Information contents:**

Details on methods

Figure S1. Audience reaction to the species featured in Planet Earth 2.

Figure S2. Audience engagement to species featured in Planet Earth 2.

Figure S3. Causal impact of Planet Earth 2 on long-term audience awareness.

Figure S4. Anomaly analysis of charity donation time series.

Figure S5. Audience engagement for information in response to Planet Earth 2 and to world species days.

Table S1. Mentions of conservation and environmental themes in Planet Earth 2.

Table S2. Audience reaction to conservation and environmental themes covered in Planet Earth 2.

Table S3. Output of negative binomial GLM models explaining audience reaction to Planet Earth 2.

Table S4. Planet Earth 2 species Wikipedia page visit anomalies.

Table S5. Output of negative binomial GLM models explaining audience engagement following Planet Earth 2.

Table S6. Summary statistics of causal impact of Planet Earth 2 on long-term audience awareness.

Table S7. Comparison of audience engagement for information in response to Planet Earth 2 and to world species days.

**Other Supporting Information for this manuscript include the following:**

Data S1. Dataset (excel file).

Data S2. R project code (online files).

**Details on methods**

**Portrayal of the natural world: Planet Earth 2**

We carried out a qualitative content analysis on the script of the show to identify conservation themes. We reviewed the episode scripts separately to avoid bias (one review per author). When we assessed both reviews jointly, we confirmed that the same sections had been identified in both reviews, with differences emerging only in the starting and ending point of each section. Final highlighted sections were the result of combining both reviews (i.e. sections starting at the earliest and ending at the latest point highlighted by either review). The percentage of the script allocated to environmental and conservation topics was calculated using the total number of words in the highlighted sections divided by the total number of words in each episode and across all episodes. We also used the highlighted sections to identify specific conservation themes covered by the show.

Species time on screen was calculated as the time elapsed from first to last appearance on screen for each episode. If the species also appeared in the ‘diaries’ making-of section at the end of the episode, we added the species’ time on screen in this section to its time on screen during the episode.

**Audience Reaction: Twitter Activity**

As some of the conservation themes we uncovered in our script analysis were mentioned in multiple episodes, we counted their occurrence across all tweets (n = 30000). We also searched for three additional themes (‘conservation’, ‘environment’, and ‘donations’) which may show effective transfer of conservation messages.

We only included species that could be unambiguously identified on Twitter. For instance, episode 1 features multiple species of lemur so we discounted mentions of ‘lemur’ on Twitter in this case because it was not clear which species was being referred to. In total, this gave us Twitter data for 94 out of the 113 species possible.

Audience engagement: Wikipedia page visits

The *anomalyDetection* packages uses Seasonal Hybrid ESD to detect anomalies in time series (described by Hochenbaum, J., Vallis, O. S., & Kejariwal, A. 2017. Automatic anomaly detection in the cloud via statistical learning. arXiv preprint arXiv:1704.07706). In assessing the anomalies, we used information from the Wikipedia page corresponding to the name of the species as it was stated in the script in its most specific form. For example, if the script used both ‘Iguana’ and ‘Marine iguana’ we searched for the latter. This gave data on Wikipedia pages for 112 of the 113 species featured in the show. As the *anomalyDetection* package distinguishes between users who accessed the website on a mobile device or a desktop, we combined these data for all of our analyses.

For the model building we also excluded 5 data points that had a negative difference (i.e. their value was less than the baseline) so that we could carry out an analysis on count data, resulting in a sample size of 107 out of a possible 112 species.

**Changes in awareness: long-term trends in Wikipedia page visits**

For our causal impact analysis, we set out a pre-period time (May – June 2016) before the show was broadcast and a post-period time after the broadcast had finished (May – June 2017). This post-period was chosen to avoid any confounding effects from the USA broadcast of the show.

As Wikipedia users and page visits may increase naturally over time and produce a false positive (i.e. increase in page visits stemming from increased Wikipedia activity rather than a Planet Earth 2 effect), we selected control time series for each model that would not be affected by the broadcast. For each target time series, we chose time series of five species featured in Planet Earth 1 (broadcast in 2006) that did not feature in Planet Earth 2. These were selected according to the similarity in median value of page hits from all the Planet Earth 1 time series.

**Signal strength**

We calculated the baseline popularity separately for each of the 16 species over an 11-month period starting two weeks after the species day in 2015 and ending two weeks before the species day in 2016 (thus avoiding any signal of the species day itself affecting our baseline measure).


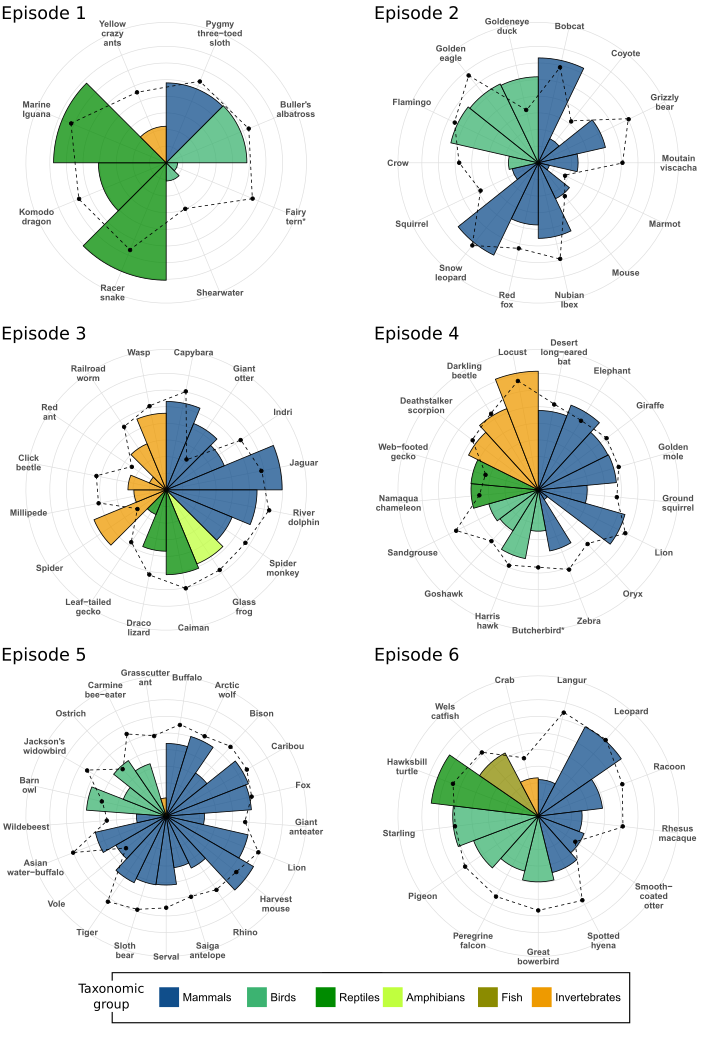


Fig. S1. Audience reaction to the species featured in Planet Earth 2.

Audience reaction was measured as the number of species mentions in tweets with the hashtag #PlanetEarth2; dashed lines represent time on screen for each species (values are log-transformed; untransformed value ranges: 9-1250 tweets, 98-824 sec). Species with no audience reaction are not represented, ‘*’ denote inaccurately named species (see Fig. 4 in main text).

*Episode 1*


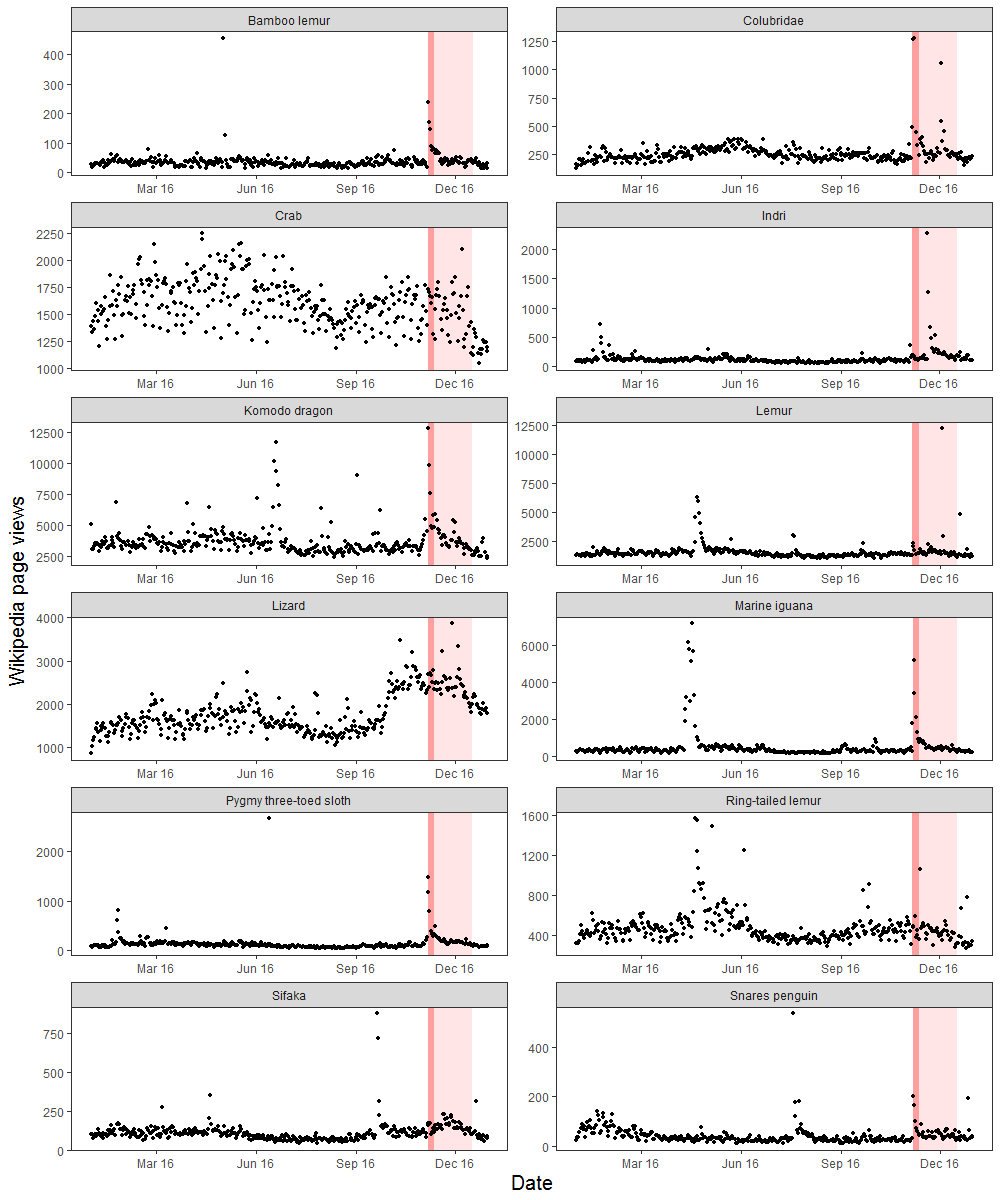


Fig. S2. Audience engagement to species featured in Planet Earth 2.

Engagement was measured as visits to the Wikipedia species pages; red shading represents the six weeks of the broadcast and dark red band represents the week of the broadcast of the corresponding episode.

(*Fig. S2 continued*)


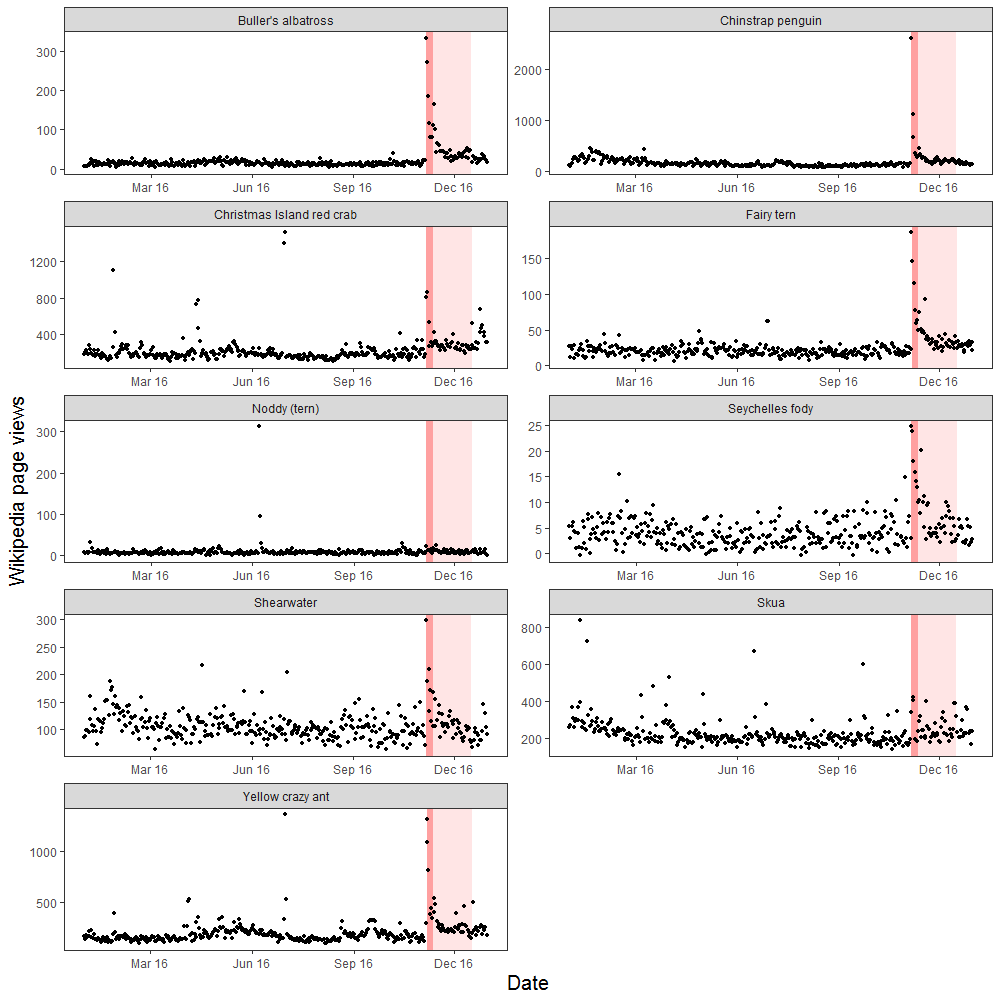


*Episode 2*


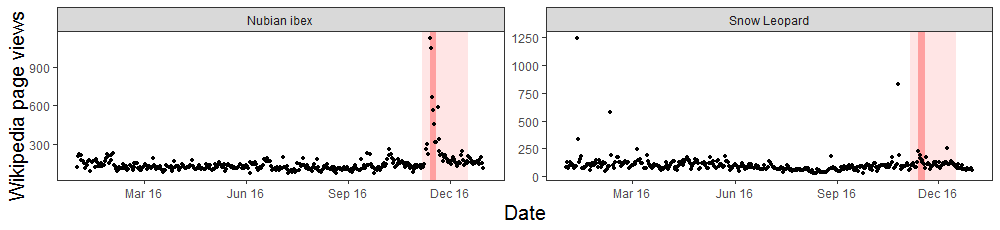


(*Fig. S2 continued*)


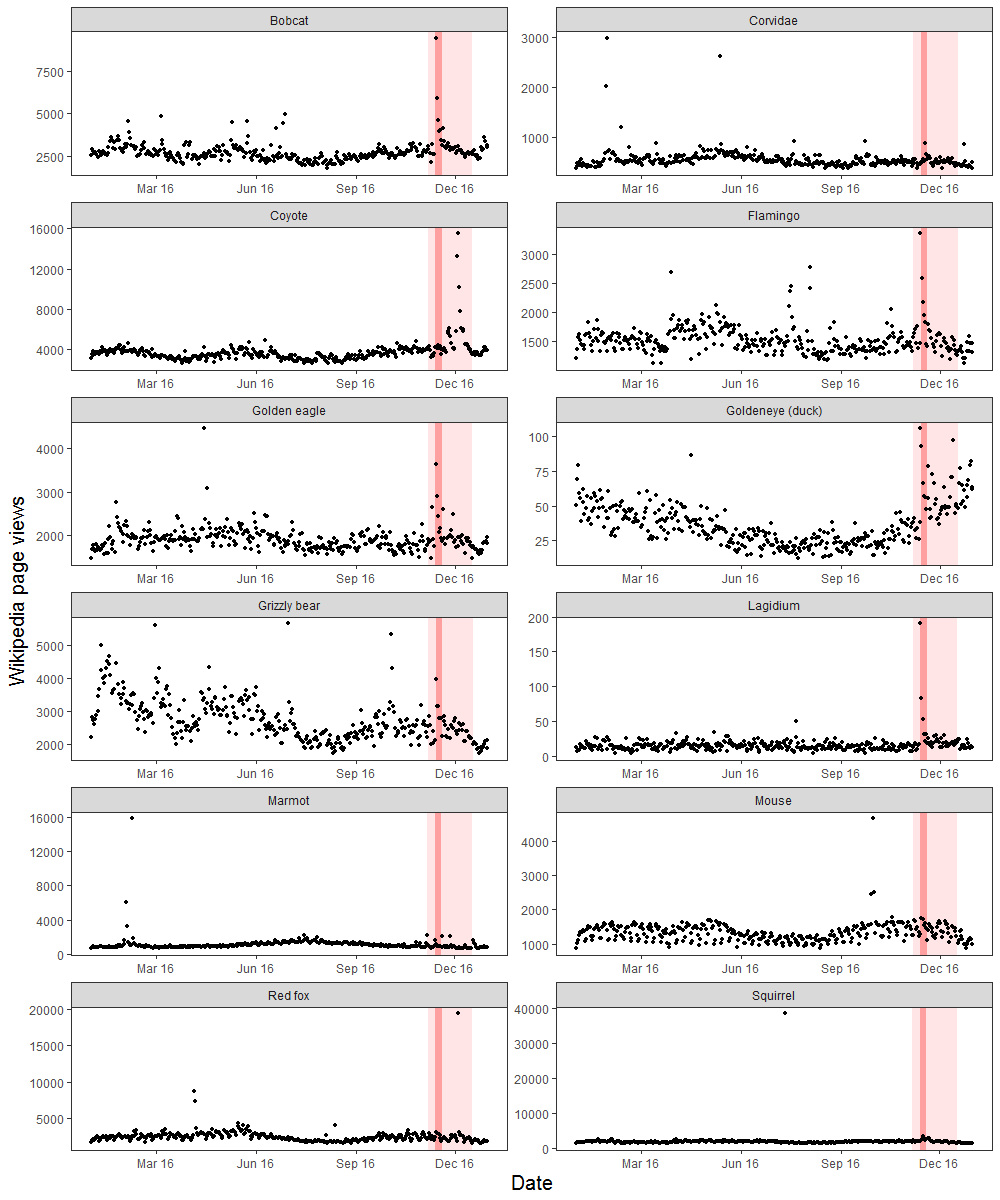


(*Fig. S2 continued*)

*Episode 3*


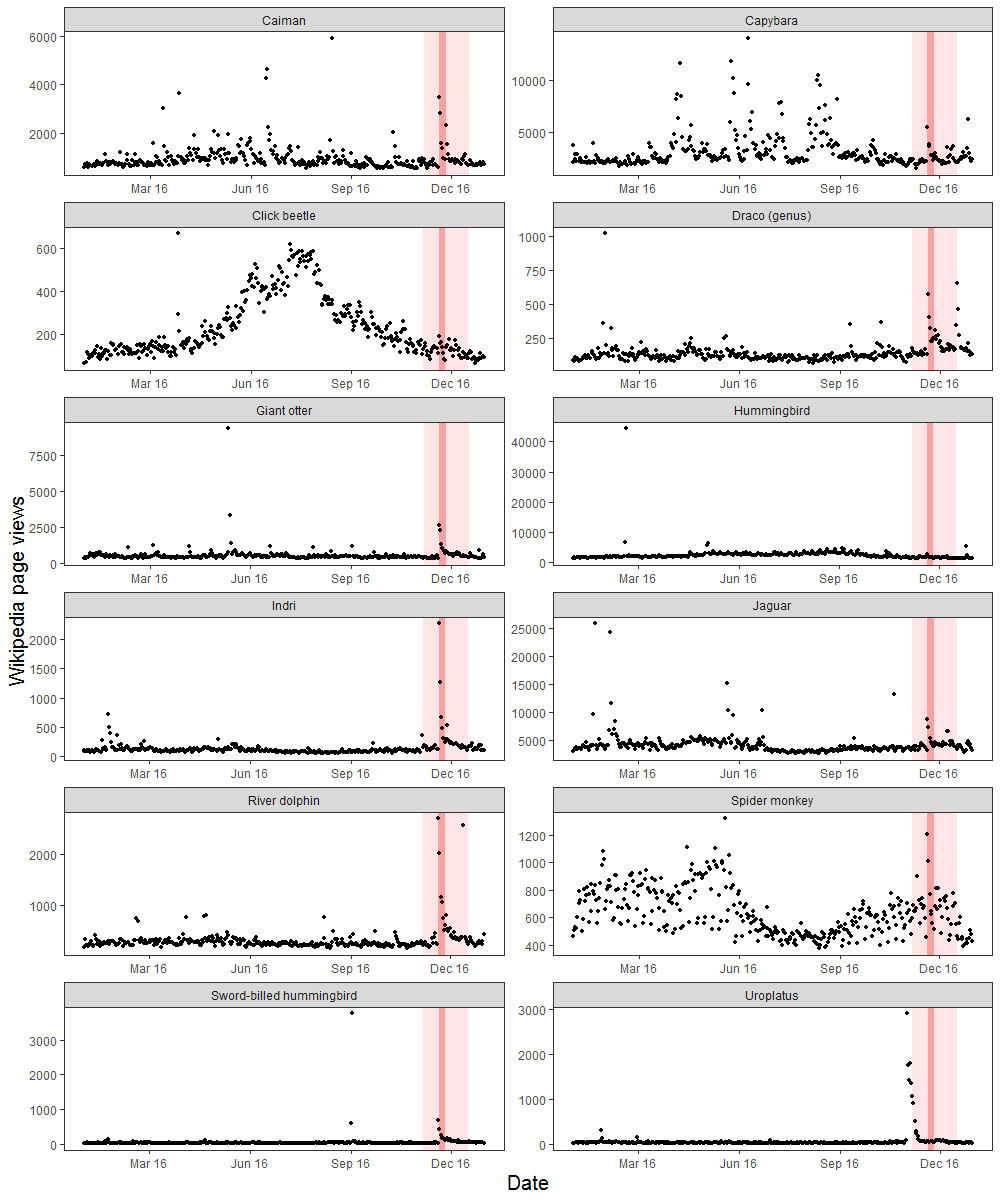


(*Fig. S2 continued*)


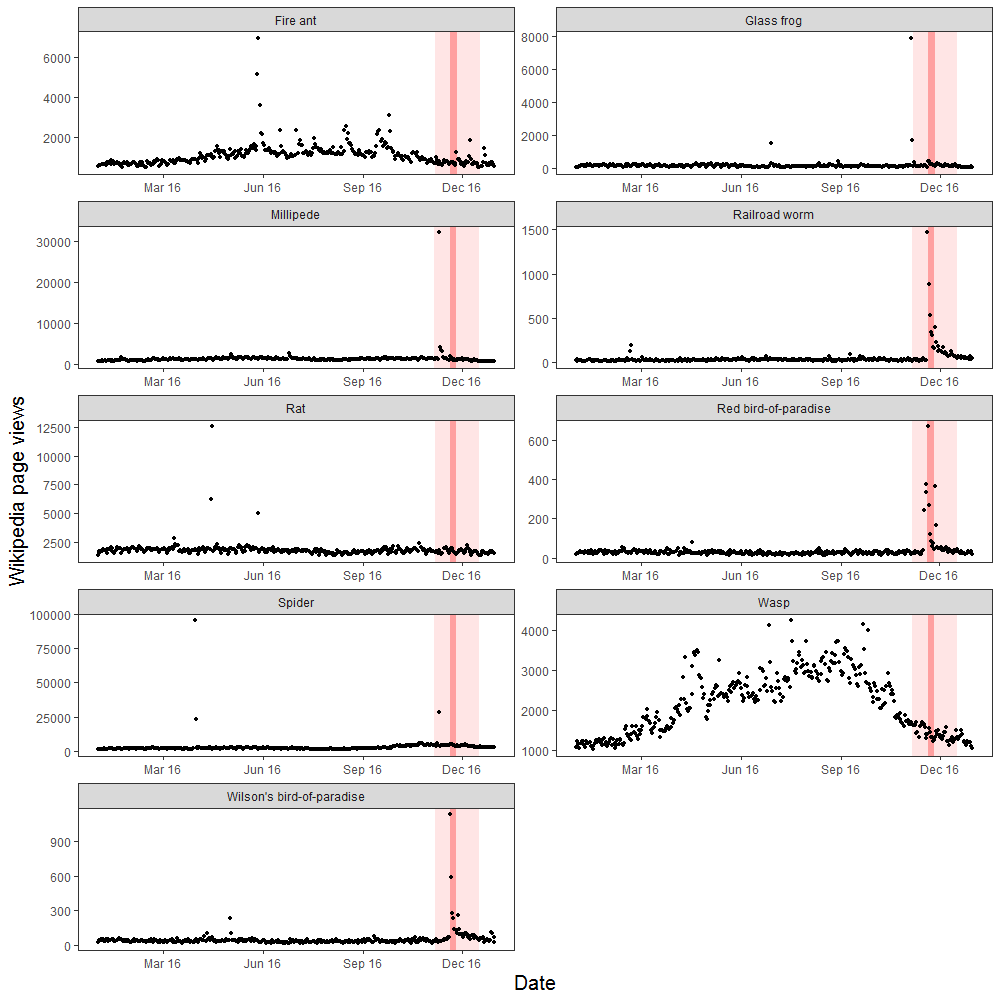


*Episode 4*


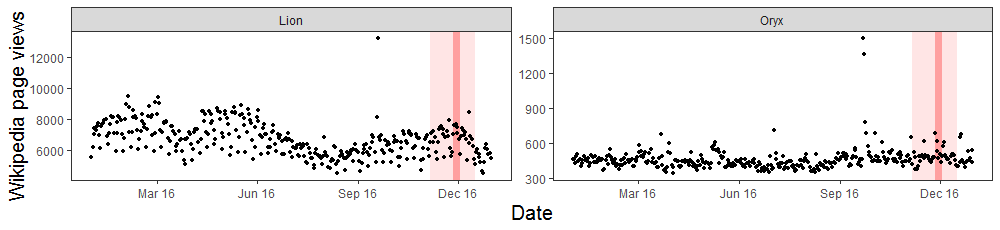


(*Fig. S2 continued*)


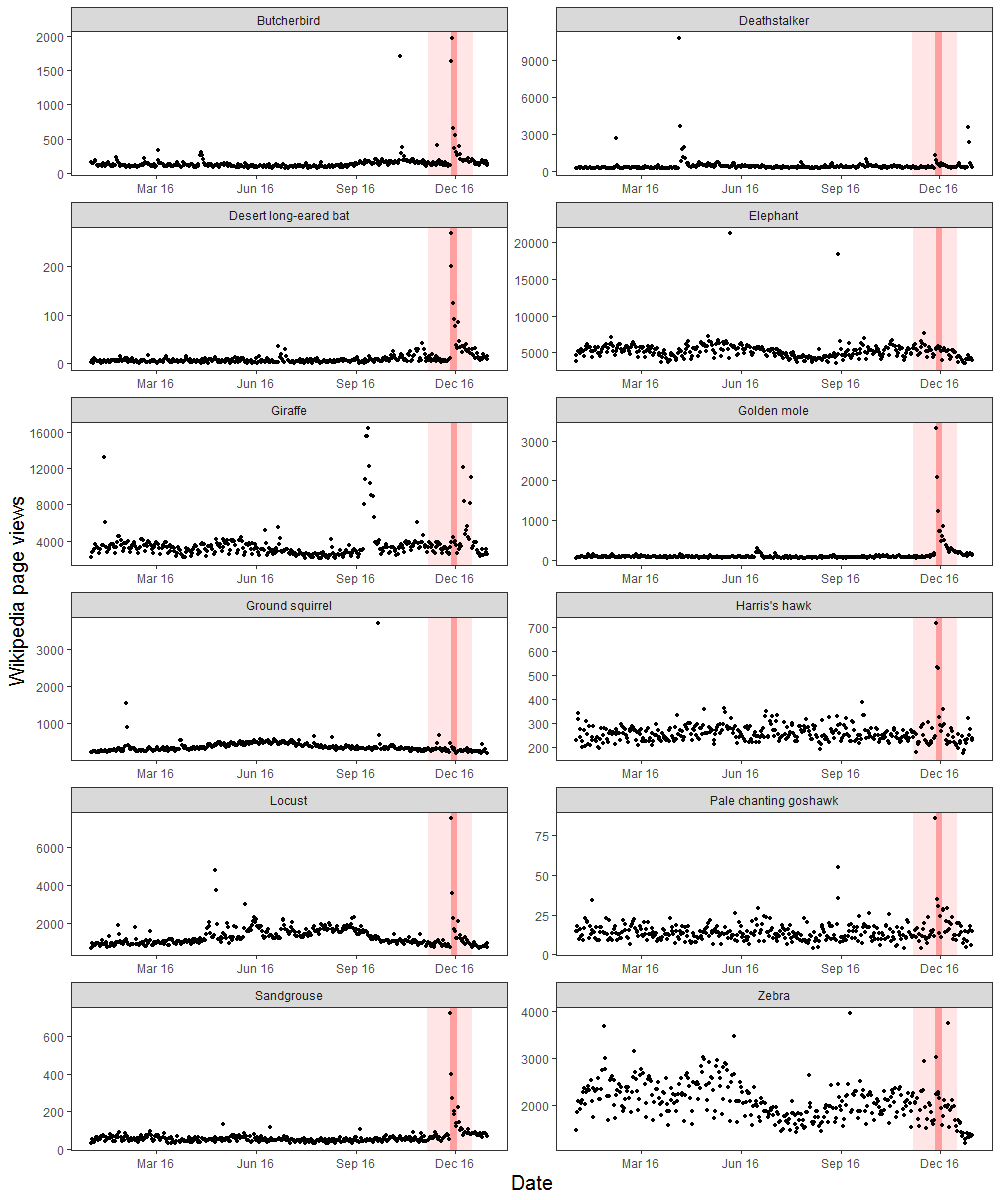


(*Fig. S2 continued*)


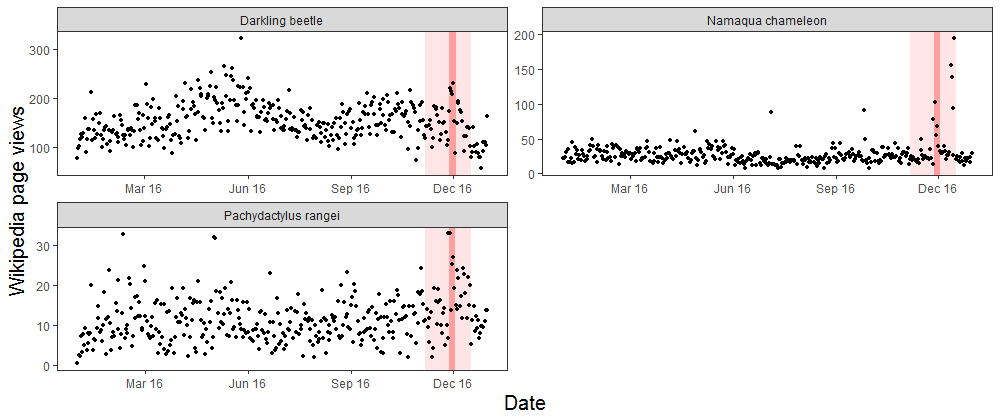


*Episode 5*


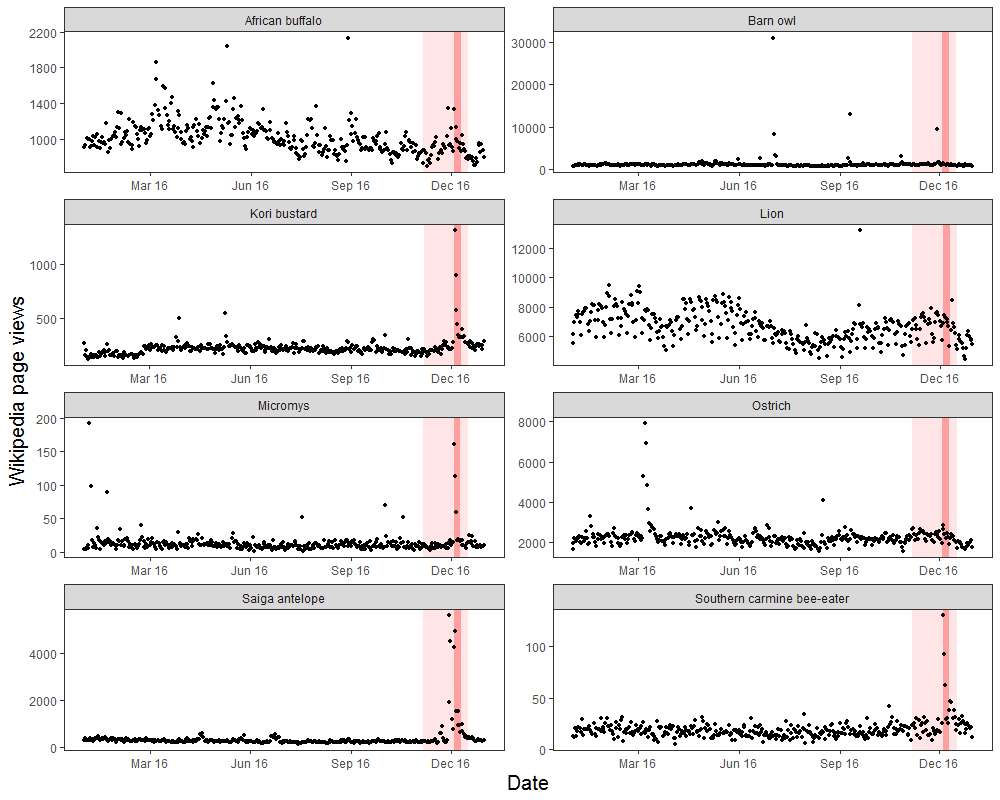


(*Fig. S2 continued*)


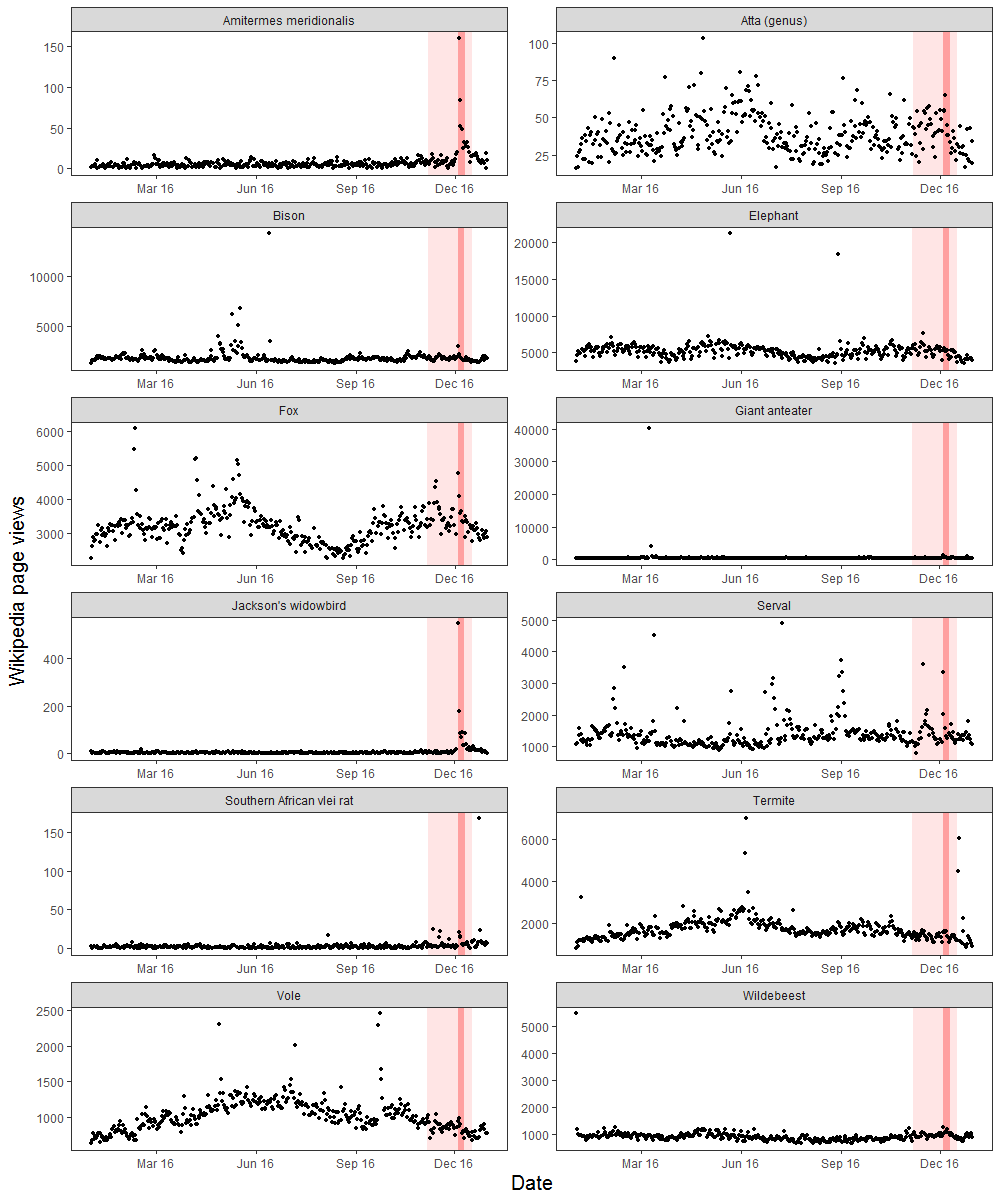


(*Fig. S2 continued*)


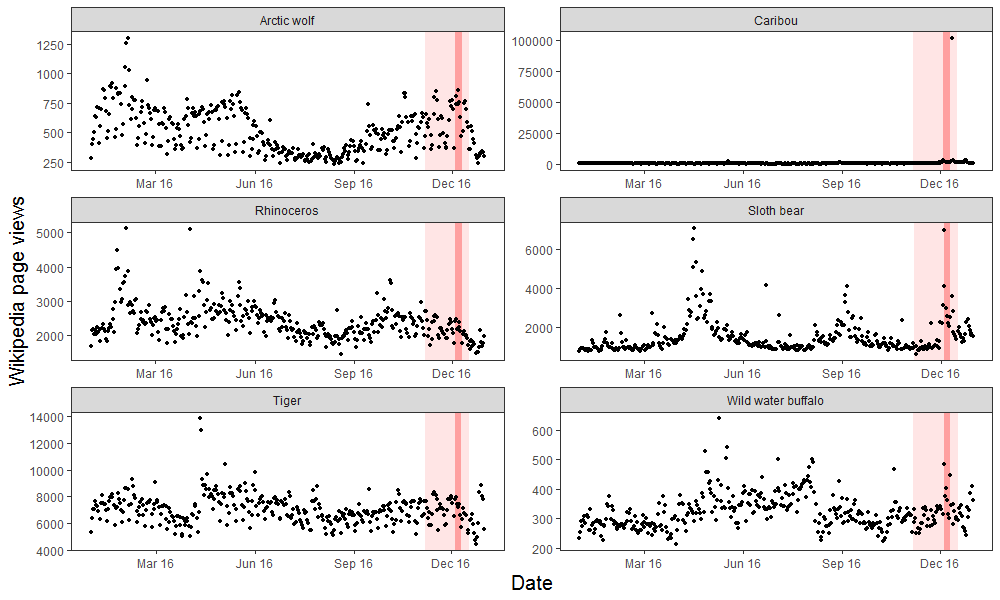


*Episode 6*


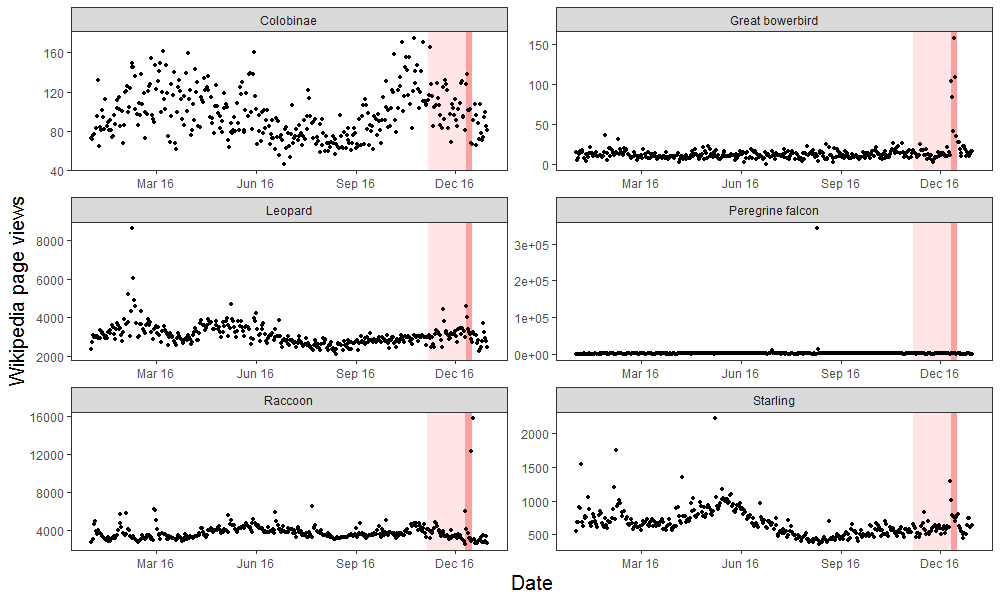


(*Fig. S2 continued*)


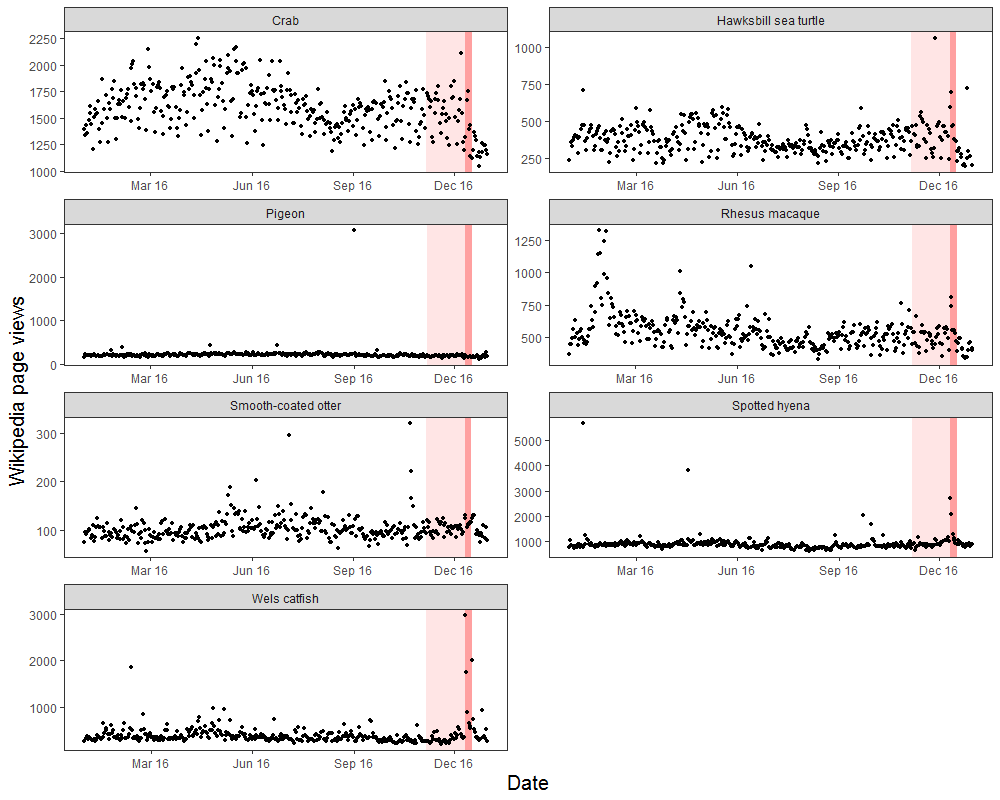


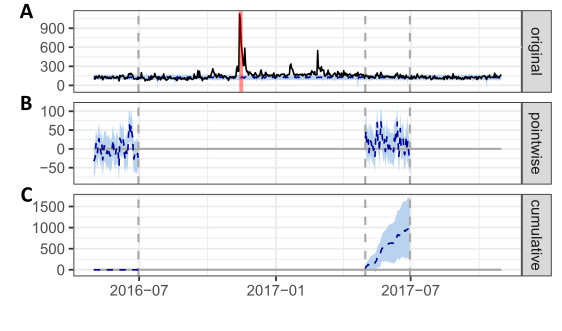
Fig. S3. Causal impact of Planet Earth 2 on long-term audience awareness.

(**A**) Time series of visits to the Wikipedia page for Nubian ibex *Capra nubiana* related to control species not featured in the show (red band indicates episode broadcast), the solid black line represents the data, the dashed blue line is the counterfactual prediction, the vertical lines indicate the pre-period and post-period. (**B**) Pointwise (daily) incremental impact of Planet Earth 2 on page visits, this is the difference between what was observed and the counterfactual prediction; (**C**) cumulative impact of Planet Earth 2 on page visits during the post-period, this is based on summing the values from the pointwise causal effect in the second panel.


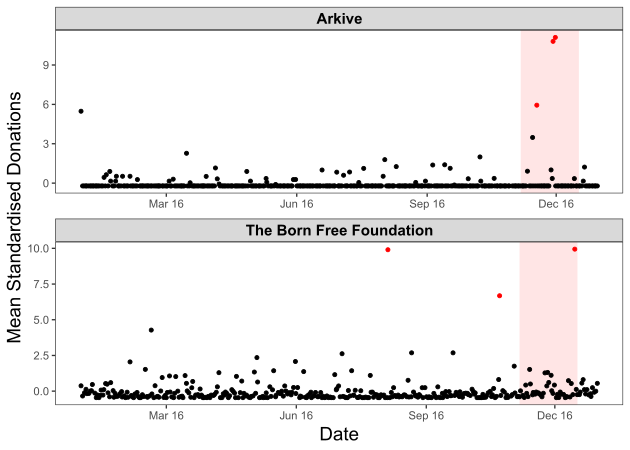
Fig. S4. Anomaly analysis of charity donation time series.

Anomaly analysis of donations to conservation charities during 2016; red dots indicate anomalies and donations are mean standardized. These data revealed no clear effect of the broadcast of Planet Earth 2. Donations to Arkive and the Born Free Foundation registered anomalies within a week after the broadcast of a Planet Earth 2 episode (three out of three yearly anomalies for Arkive, one out of three yearly anomalies for the Born Free Foundation). However, it was not possible to establish a clear causal effect with the show as these anomalies were outside the threshold we had set (on the day or the day after an episode broadcast). The closest anomaly was two days after the broadcast of an episode.


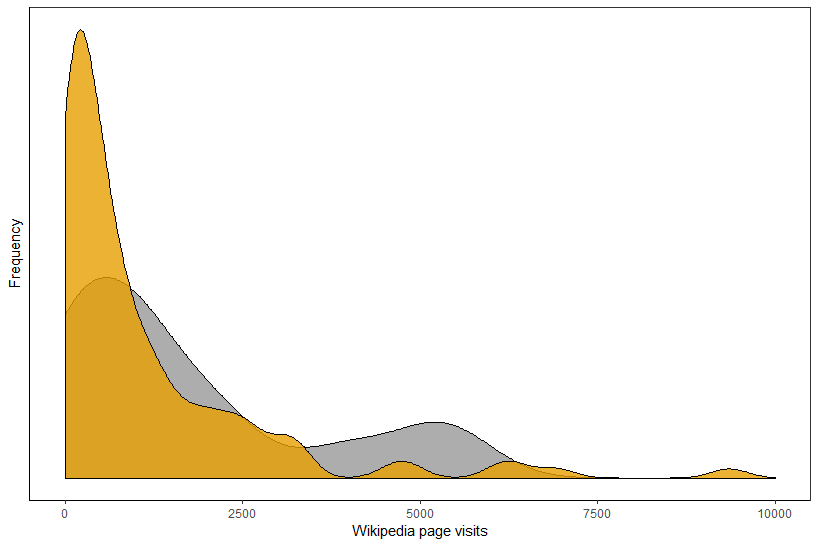
Fig. S5. Audience engagement for information in response to Planet Earth 2 and to world species days.

Frequency distribution of Wikipedia page visits for all species featured in Planet Earth 2 (orange) compared to page visits for 16 world species days (grey; listed in Table S7). Wikipedia page visits were calculated as the number of visits on broadcast days or world species days minus the species’ baseline popularity (see methods).

Table S1. Mentions of conservation and environmental themes in Planet Earth 2.

Number of mentions of conservation and environmental themes in each episode of Planet Earth 2 and percentage of the script dedicated to these topics.

| **Episode** | **Number of mentions** | **Percentage of script** |
| --- | --- | --- |
| 1: Islands | 6 | 7.4% |
| 2: Mountains | 1 | 4.0% |
| 3: Jungles | 1 | 2.2% |
| 4: Deserts | 2 | 2.3% |
| 5: Grasslands | 0 | 0% |
| 6: Cities | 6 | 21.0% |
| All episodes | 16 | 6.4% |

Table S2. Audience reaction to conservation and environmental themes covered in Planet Earth 2.

Audience reaction was measured as the number (and percentage) of tweets with the #PlanetEarth2 which mentioned each theme, out of the collated sample of tweets (n = 30000). ‘*’ denote additional themes, not specifically mentioned in the show, that may indicate effective transfer of conservation messages.

| **Theme** | **Number of mentions** | **Percentage of tweets** |
| --- | --- | --- |
| Extinction | 19 | 0.06% |
| Climate change | 55 | 0.18% |
| Endangered | 13 | 0.04% |
| Invasive species | 2 | 0.01% |
| Desertification | 0 | 0% |
| Urbanization | 0 | 0% |
| Pollution (light) | 11 | 0.04% |
| City greening | 17 | 0.06% |
| Conservation* | 49 | 0.16% |
| Environment* | 55 | 0.18% |
| Donation* | 13 | 0.04% |

Table S3. Output of negative binomial GLM models explaining audience reaction to Planet Earth 2.

Response variables were the number of Twitter mentions for each species, explanatory variables were species time on screen, taxonomic group (baseline category was ‘amphibian’, conservation status (baseline category was ‘critically endangered’), and appearance in the diaries section. Ecological interaction portrayed in the show (predator-prey or other) was removed to remedy non-convergence. The univariate model (Cox-Snell Pseudo R Squared = 0.372) was preferred over the full model based on AIC.

| **Model** | **Factor** | **Estimate** | **SE** | **z value** | **p-value** |
| --- | --- | --- | --- | --- | --- |
| Univariate | Intercept | 3.478 | 0.198 | 17.562 | 0.000 |
| (df = 3, AIC = 1045.0) | Time on screen | 0.005 | 0.001 | 7.06 | 0.000 |
| Full | Intercept | 3.289 | 1.277 | 2.610 | 0.009 |
| (df = 14, AIC = 1057.8) | Time on screen | 0.005 | 0.001 | 5.535 | 0.000 |
|  | Taxa: Birds | 0.005 | 1.181 | 0.005 | 0.996 |
|  | Fish | 0.236 | 1.624 | 0.145 | 0.885 |
|  | Invertebrate | 0.890 | 1.291 | 0.670 | 0.490 |
|  | Mammal | 0.248 | 1.172 | 0.211 | 0.833 |
|  | Reptile | 1.367 | 1.236 | 1.114 | 0.265 |
|  | Status: EN | -0.118 | 0.734 | -0.161 | 0.872 |
|  | VU | 0.113 | 0.602 | 0.188 | 0.851 |
|  | NT | 0.160 | 0.652 | 0.246 | 0.806 |
|  | LC | -0.019 | 0.567 | -0.033 | 0.973 |
|  | DD | -0.562 | 0.679 | -0.826 | 0.409 |
|  | Diaries | -0.537 | 0.418 | -1.286 | 0.199 |

Table S4. Planet Earth 2 species Wikipedia page visit anomalies.

Number (and percentage, in brackets) of Planet Earth 2 species that registered yearly 2016 anomalies in Wikipedia page visits on broadcast days (day of and day after broadcast) compared to ‘control’ Planet Earth 1 species during the same time period. Significantly more Planet Earth 2 species registered anomalies than control species (Chi square = 38.792, df =1, p < 0.001).

| **Species** | **Peaks on one broadcast day** | **Peaks on both broadcast days** |
| --- | --- | --- |
| Planet Earth 2 | 46 (41.1%) | 25 (22.3%) |
| Control (Planet Earth 1) | 8 (6.6%) | 1 (0.8%) |

Table S5. Output of negative binomial GLM models explaining audience engagement following Planet Earth 2.

Response variables were the number of Wikipedia page visits corrected for baseline popularity (see methods) for each species, explanatory variables were species time on screen, taxonomic group (baseline category was ‘amphibian’, conservation status (baseline category was ‘critically endangered’), ecological interaction portrayed in the show (baseline category was ‘other’), and appearance in the diaries section. The univariate model (Cox-Snell Pseudo R Squared = 0.174) was preferred over the full model based on AIC.

| **Model** | **Factor** | **Estimate** | **SE** | **z value** | **p-value** |
| --- | --- | --- | --- | --- | --- |
| Univariate | Intercept | 6.285 | 0.171 | 36.771 | 0.000 |
| (df = 3, AIC = 1688.9) | Time on screen | 0.003 | 0.001 | 4.781 | 0.000 |
| Full | Intercept | 5.393 | 1.238 | 4.357 | 0.000 |
| (df = 15, AIC = 1700.3) | Time on screen | 0.002 | 0.001 | 3.088 | 0.002 |
|  | Taxa: Birds | 0.840 | 1.164 | 0.721 | 0.471 |
|  | Fish | 2.561 | 1.586 | 1.615 | 0.106 |
|  | Invertebrates | 1.849 | 1.246 | 1.484 | 0.138 |
|  | Mammal | 1.418 | 1.152 | 1.231 | 0.218 |
|  | Reptile | 1.848 | 1.212 | 1.525 | 0.127 |
|  | Status: EN | -0.773 | 0.604 | -1.280 | 0.201 |
|  | VU | -0.204 | 0.533 | -0.383 | 0.702 |
|  | NT | 0.113 | 0.561 | 0.201 | 0.841 |
|  | LC | -0.356 | 0.494 | -0.720 | 0.471 |
|  | DD | -0.867 | 0.587 | -1.476 | 0.140 |
|  | Interaction: pred./prey | -0.120 | 0.285 | -0.420 | 0.675 |
|  | Diaries | 0.106 | 0.403 | 0.265 | 0.791 |

Table S6. Summary statistics of causal impact of Planet Earth 2 on long-term audience awareness.

We summarized the results of our causal impact analysis by compiling the posterior tail-area probabilities for each of the species. This value is the probability of seeing a value as extreme as the one recorded if no intervention had occurred (in our case, if Planet Earth 2 had not been aired). Of the 43 species tested 19 (44%) had a positive absolute effect with a posterior tail-area probability less than 0.05, suggestive that there is a long-term effect on these species when using Planet Earth 1 species as a control. The average absolute effect shows the causal impact of Planet Earth 2 on each article (CI = credible interval).

| **Article names** | **95% CI (lower)** | **Average absolute effect** | **95% CI (upper)** | **Posterior probability** |
| --- | --- | --- | --- | --- |
| *Amitermes meridionalis* | 4.78 | 6.26 | 7.74 | 0.001 |
| Bamboo lemur | -33.50 | -7.19 | 21.69 | 0.288 |
| Bobcat | -428.00 | -131.64 | 170.59 | 0.189 |
| Buller’s albatross | 1.27 | 3.91 | 6.69 | 0.004 |
| Butcherbird | 73.77 | 81.71 | 90.62 | 0.001 |
| Caribou | -101.40 | 11.19 | 136.61 | 0.455 |
| Chinstrap penguin | 6.32 | 20.76 | 36.51 | 0.004 |
| Colubridae | 5.71 | 26.12 | 50.92 | 0.007 |
| Desert long-eared bat | 4.83 | 7.79 | 10.94 | 0.001 |
| *Draco genus* | 0.05 | 18.07 | 36.98 | 0.026 |
| Fairy tern | -1.88 | 2.31 | 6.66 | 0.17 |
| Flamingo | 199 | 294.27 | 395.40 | 0.001 |
| Giant anteater | -17.95 | 13.30 | 49.07 | 0.217 |
| Giant otter | -624.53 | -86.29 | 492.12 | 0.397 |
| Golden eagle | -241.72 | -150.23 | -46.79 | 0.004 |
| Golden mole | -11.21 | 10.77 | 31.22 | 0.177 |
| Goldeneye duck | -0.63 | 2.94 | 6.54 | 0.062 |
| Great bowerbird | 1.61 | 3.48 | 5.56 | 0.002 |
| Harris’s hawk | -12.12 | 2.90 | 20.00 | 0.370 |
| Komodo dragon | -1110.33 | -280.98 | 562.55 | 0.246 |
| Kori bustard | -25.09 | 0.40 | 25.55 | 0.499 |
| *Lagidium* | -1.88 | 0.99 | 3.94 | 0.251 |
| Locust | -521.85 | -344.48 | -160.05 | 0.001 |
| *Micromys* | -0.81 | 1.45 | 3.78 | 0.106 |
| Nubian ibex | 3.23 | 15.17 | 27.97 | 0.009 |
| *Pachydactylus rangei* | -0.65 | 2.18 | 5.19 | 0.065 |
| Pale chanting goshawk | -1.25 | 1.17 | 3.68 | 0.184 |
| Pygmy three-toed sloth | -201.31 | -39.47 | 129.55 | 0.314 |
| Railroad worm | 7.29 | 11.83 | 16.41 | 0.002 |
| Red bird of paradise | 2.32 | 5.27 | 8.67 | 0.001 |
| River dolphin | 21.55 | 49.87 | 81.97 | 0.001 |
| Saiga antelope | -13.62 | 28.82 | 74.94 | 0.102 |
| Sandgrouse | 5.83 | 13.33 | 21.16 | 0.001 |
| Shearwater | -6.98 | 1.60 | 10.73 | 0.368 |
| Sloth bear | -884.74 | -507.32 | -157.04 | 0.006 |
| Snares penguin | 1.29 | 5.39 | 9.95 | 0.008 |
| Southern carmine bee-eater | 3.35 | 5.52 | 7.56 | 0.001 |
| Spider monkey | -151.16 | -39.52 | 86.07 | 0.237 |
| Spotted hyena | 65.33 | 113.3 | 165.3 | 0.001 |
| Sword-billed hummingbird | 7.89 | 12.33 | 17.19 | 0.001 |
| Wels catfish | -15.38 | 42.27 | 102.7 | 0.081 |
| Wilson’s bird of paradise | 7.75 | 21.42 | 36.3 | 0.003 |
| Yellow crazy ant | -15.02 | 8.02 | 32.32 | 0.256 |

Table S7. Comparison of audience engagement for information in response to Planet Earth 2 and to world species days.

Engagement for information was measured as Wikipedia page visits corrected for baseline popularity (i.e. page visits on broadcast days or on world species days minus prior median page visits, see methods). 22% of all species featured in Planet Earth 2 (and 34% of those that registered an anomaly) had more Wikipedia page visits than the mean for species on the dates of their corresponding world species day. Negative values indicate that, for some species, page visits on broadcast or world species day were below the median.

| **Species** | **Wikipedia page visits** |
| --- | --- |
| Dolphin | 1021 |
| Giant panda | 989 |
| Giraffe | 2079 |
| Honey bee | 162 |
| Lion | -153 |
| Orangutan | -130 |
| Pangolin | 5053 |
| Polar bear | 5558 |
| Rhinoceros | 1160 |
| Sea turtle | 1163 |
| Sloth | 3896 |
| Snake | 91 |
| Tiger | 2247 |
| Vulture | 149 |
| Whale | 20 |
| Whale shark | 427 |
| Planet Earth 2 species mean (and range) | 1071 (-153, 5558) |
| World species day mean (and range) | 1483 (-546, 9360) |

Data S1. (separate file)

Dataset (excel format) used for Planet Earth 2 conservation knowledge transfer analysis. This includes species information for the different steps of the framework: natural world (scientific name, taxa, IUCN status); Planet Earth 2 (name used in script, episode, time on screen, appearance in diaries, ecological interaction portrayed, comments on data collection); audience reaction (twitter search word, number of Twitter mentions); audience engagement for information (species Wikipedia page, baseline popularity, visits on broadcast days, visits attributed to Planet Earth 2 audiences, anomalies).

Data S2. (online files)

R project and code for all analysis is available at https://github.com/kanead/documentary-paper.
